# Health-Promoting Leadership: Concept, Measurement, and Research Framework

**DOI:** 10.3389/fpsyg.2021.602333

**Published:** 2021-02-26

**Authors:** Lei Yao, Ping Li, Helen Wildy

**Affiliations:** ^1^Business School, Beijing Normal University, Beijing, China; ^2^Center for Business Ethics Studies, Henan University of Economic and Law, Zhengzhou, China; ^3^Faculty of Arts, Business, Law and Education, Graduate School of Education, University of Western Australia, Perth, WA, Australia

**Keywords:** health-promoting leadership, workplace health, leadership, health, well-being

## Abstract

Employee health is not only positively related to the employee well-being and family happiness, but also impacts organizations, and society as a whole. We searched the health-promoting leadership literature in the following databases: Web of Science, ProQuest, EBSCO, and a Chinese local database. Based on this research, we clarify the concept of health-promoting leadership, propose a definition of health-promoting leadership, and examine measurement scales for this type of leadership. We also suggest a research framework for health-promoting leadership, demonstrating its potential outcomes at both the individual level (e.g., health, well-being, job attitudes) and the organizational level (e.g., health management culture and practices); the mechanisms for its development based on conservation of resources theory, the job demands–resources model, social learning theory, and social exchange theory; and antecedents (e.g., health values, health awareness, organizational health culture, organizational health climate, and organizational health promotion behavior control). Finally, we identify six potential research areas: Research level, performance, the impacts of health-promoting leaders on themselves, moderators, research methods, and intervention effects on health-promoting leadership.

## Introduction

Health is essential for human survival and sustainable development. Unfortunately, within the competitive environment in the workplace, mental health issues such as burnout, anxiety, depression, and suicide are becoming increasingly severe. At the same time, serious incidents caused by neglect of employees' physical and mental health are being more frequently reported. Organizations are urged to pay attention to employee health issues and shoulder the responsibility for promoting and assuring employee health for a multitude of reasons (Rigotti et al., 2014; Horstmann and Eckerth, 2016). Notably, employee health is not only related to employees' well-being, but also closely related to organizational performance (Hennekam et al., 2020; Salas-Vallina et al., 2020). In addition, research has found that employee health is positively related to the organization's explicit costs (e.g., medical insurance expenses) and hidden costs (e.g., absenteeism due to illness) (Gurt et al., 2011), which affect organizational sustainable development (Pescud et al., 2015). Organizations are therefore advised to respond actively and proactively to employee health issues. In this social context, the concept of health-promoting leadership has emerged as a salient factor: It not only expands the traditional leadership theory, but also has practical implications for confronting the tricky issues of employee health.

Although scholars consistently agree that health-promoting leadership has an important influence on organizational development and employee well-being, some important issues still need to be systematically clarified. For example, what are the key components of health-promoting leadership? Which research has been carried out on this type of leadership? Which direction should future research take? To answer these questions, we summarize the research on health-promoting leadership from five aspects: concept, structure and measurement, consequences, mechanism, and antecedents. This study further proposes a research framework and indicates six promising research directions to deepen the understanding of health-promoting leadership.

## Method

### Literature Research

We searched the health-promoting leadership literature published from 2010 to 2020 in the following databases: Web of Science, ProQuest, EBSCO, and a Chinese local database. To perform the search, we used the keywords “health-promoting leadership,” “healthy leadership,” “health-oriented leadership,” “health-specific leadership,” and “health-relevant leadership.” We also used a snowball approach by searching the references lists in the research we found in an effort to identify related literature.

### Study Inclusion and Exclusion Criteria

Researches were included in the review if they met the following inclusion criteria. First, we included research for which the topic was health-promoting leadership and excluded leadership research that did not directly pertain to health. Second, we classified authors' work that had been published in both journal and dissertation form as the same research. Third, the publications had to be written in English or Chinese.

**Figure 1:**
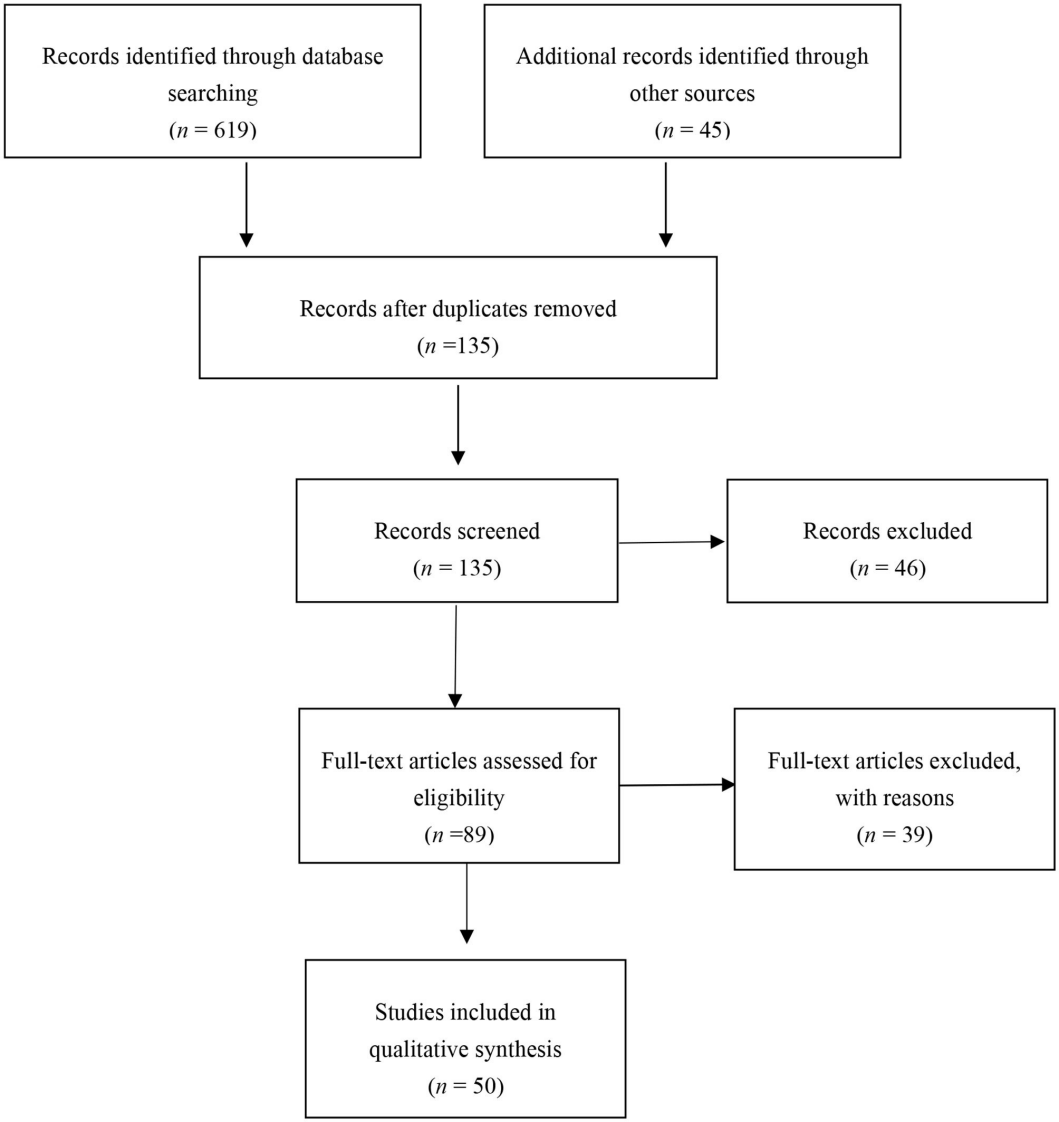
Screening process in accordance with PRISMA guidelines.

We included both quantitative and qualitative research, and did not place any constraints on the locations in which the research was conducted. Participants included government officials as well as staff in business organizations in fields such as healthcare, education, IT and telecommunication, manufacturing, and commerce. We also included published articles, conference papers, working papers, and doctoral dissertations. Ultimately, we ended up with 50 studies. Figure 1 summarizes our search process.

## The Concept of Health-Promoting Leadership

The concept of health-promoting leadership has received much attention from scholars, with authors adopting either a general perspective or a specific perspective to address it. According to our literature review, the general perspective definition is based on traditional leadership behaviors and focuses on the positive effects of these behaviors on employee health. From this perspective, health-promoting leadership encompass general good leader behaviors that promote employees' health (e.g., individualized care). For example, Eriksson et al. (2012) demonstrate that health-promoting leadership comes from “supporting good leadership in general and improving general working conditions of managers.” Winkler et al. (2014) suggest that leaders promote employees' health through supportive behaviors such as individual consideration and positive feedback. In their work, Vincent-Höper and Stein (2019) used the job demands–resources model to divide traditional leadership behaviors related to stress into three types: demanding leadership behavior, support-oriented leadership behavior, and development-orientated leadership.

Some scholars have subsequently adopted both general good leadership and specific health-promoting behaviors to define health-promoting leadership. Gurt and Elke (2009) indicated that health-promoting leadership includes both general good leadership (e.g., recognizing employees' achievements) and specific health-promoting leadership behaviors (e.g., encouraging employees to participate health-promoting projects). Eriksson et al. (2011) used a phenomenological approach to describe health-promoting leadership as both showing traditional leadership behaviors that support employees and developing a healthy work environment by implementing health-oriented interventions. We could see that the definition of health-promoting leadership from the general perspective is based on the traditional understanding of good leadership.

However, empirical research has also found that general good leadership can potentially harm employees' health (Barling et al., 2002). For example, Nielsen and Daniels (2016) demonstrated that transformational leadership has a negative impact on employees who are sick and may increase their absenteeism rate after the first year of such leadership. Moreover, engaging leadership, an emergent leadership style in the literature, tends to increase employees' work engagement (Rahmadani et al., 2020)—but employees with high work engagement may then report having a high workload. Thus, engaging leadership may potentially strengthen the relationship between work overload and employees' negative health symptoms (Britt et al., 2005).

In contrast to the general perspective, the specific perspective adopted in the literature focuses on the unique impacts of health-promoting leaders on employee health. Health-promoting leaders change employees' health awareness, health motivation, and health behaviors through their own health awareness, health motivation, and health behavior (Franke et al., 2014). Such leaders also seek to cultivate a health-promoting culture and climate (Horstmann and Eckerth, 2016). In other words, health-promoting leadership takes health responsibility for employees (Gurt et al., 2011). For example, Franke et al. (2014) indicated that health-promoting leaders believe that this kind of leadership can change employees' health awareness, health values, and health behaviors through daily management and role modeling behaviors. Their concept of health-promoting leadership has been widely used in many empirical research studies (Franke et al., 2014; Horstmann, 2018a; Kaluza et al., 2018; Köppe et al., 2018). In addition, Horstmann and Eckerth (2016) proposed that employee health is a more important objective than performance and customer service under this leadership rubric.

Table 1 summarizes the two perspectives' definitions of health-promoting leadership, distilling them to their essence. The definition proposed by the specific perspective is more directly and effectively linked to employee health, so more scholars tend to use this perspective rather than the general perspective.

**Table 1:** Summary of health promoting leadership.

**Conceptual perspective**	**Core features**	**Mediator**	**Outcomes**	**Publication type**	**References**
General perspective	Good leadership, specific health-promoting behavior	Organizational health culture	Irritation, strain	Quantitative	Gurt and Elke, 2009
	Personalized care, leadership-employee positive relationship, healthy working environment, healthy behavior intervention			Qualitative	Eriksson et al., 2011
	General good leadership			Qualitative	Eriksson et al., 2012
	General good leadership		Work related well-being, job satisfaction, emotional exhaustion, psychosomatic complaints	Quantitative	Winkler et al., 2014
	Democratic and supportive leadership			Qualitative	Skarholt et al., 2016
	Demanding leadership, support-oriented leadership behavior, development-oriented leadership behavior	Job demands, job resources	Work engagement, well-being, occupational self-efficacy, irritation, emotional exhaustion	Quantitative	Vincent-Höper and Stein, 2019
Specific perspective	Health responsibility	Health climate, role ambiguity, job satisfaction	Irritation	Quantitative	Gurt et al., 2011
	Health-promoting climate	Workplace health program policies and programs, company commitment to health promotion	Job satisfaction, exhaustion/burnout, and workplace conflict	Quantitative	Milner et al., 2015
	Health responsibility			Qualitative	Larsson et al., 2015
	Health awareness, health environment		Health awareness	Quantitative	Jiménez et al., 2016
	Health awareness, health value	Self-care	Irritation, health complains, health, work-family conflict	Qualitative	Franke et al., 2014
	Health value, health awareness	Self-care	Health, job characteristic, work climate, over commitment	Qualitative	Liu, 2016
	Health responsibility, health goals/climate			Qualitative	Horstmann and Eckerth, 2016
	Health culture and value		Health promoting leadership culture	Quantitative	ŽiŽek et al., 2017
	Healthy behavior intervention	Leader health mindset	Health promoting leadership	Quantitative	Kaluza et al., 2018
	Health responsibility			Qualitative	Furunes et al., 2018
	Healthy behavior intervention		Health promoting leadership	Qualitative	Turgut et al., 2019
	Healthy behavior intervention		Safety compliance, safety proactivity	Qualitative	Nielsen et al., 2019
	Healthy behavior intervention	Social resource, task resource	Social resources, task resources	Qualitative	Bregenzer et al., 2019

## Measurement of Health-Promoting Leadership

We now examine the structure and measurement scales for health-promoting leadership, according to the definitions proposed by the general and specific perspectives (see Table 2). Currently, the measurement for the general perspective concept remains in its infancy. Eriksson (2011) explored the structural dimensions of health-promoting leadership from a theoretical perspective but did not develop a measurement scale. Vincent-Höper and Stein (2019) developed the Health- and Development-Promoting Leadership Behavior Questionnaire (HDLBQ), which includes three dimensions: demanding leadership behaviors (6 items, α = 0.64, sample item: the leader “often put me under time pressure”), support-oriented leadership behaviors (19 items, α = 0.87, sample item: the leader “supports me in the work process when I have difficulties”), and development-oriented leadership behaviors (10 items, α = 0.93, sample item: the leader “assigns me tasks which require me to use various skills and capabilities”).

**Table 2:** Summary of measurements and structures.

**Perspective**	**References**	**Dimension**	**Country**
General perspective	Gurt and Elke (2009), Eriksson et al. (2011)	Two dimensions: relation; task Three dimensions: organize health management activities, supportive leadership behaviors, and develop health management organizations	Germany Sweden
	Vincent-Höper and Stein (2019)	Three dimensions: demand-oriented leadership, support-oriented leadership, development-oriented leadership	Germany
Specific perspective	Franke et al. (2014)	Three dimensions: health awareness, health values, health behaviors	Germany
	Jiménez et al. (2016)	Seven dimensions: health awareness, workload, work control, reward, work community, value, fairness	Austria

Compared with the general perspective, the measurement tools for the specific perspective concepts are much richer. According to the scale development method, these instruments can be divided into three types.

First, researchers can adopt other constructs to measure health-promoting leadership. For example, Gurt and Elke (2009) selected seven items from the Organizational Health and Safety Scale (Gurt et al., 2010) to measure health-promoting leadership. The resulting scale is divided into two dimensions: task and relationship. A sample item for the relationship dimension is “My supervisor assumes responsibility for my health” (α = 0.91). A sample item for the task dimension is “My supervisor routinely discusses with me which objectives are to be accomplished concerning workplace health promotion” (α = 0.92). Subsequently, Gurt et al. (2011) used the scale to examine the relationship between health-promoting leaders and employees' anger.

Second, researchers can directly develop their own scale based on the concept of health-promoting leadership. Franke et al. (2014) developed a scale with three dimensions: health values (3 items; α = 0.84), health awareness (5 items; α = 0.88), and health behaviors (7 items; α = 0.84) (Franke et al., 2014). Health values refers to the degree to which individuals pay attention to health, and a sample item is “It is important for my supervisor to reduce health risks at my workplace.” Health awareness refers to the extent of the leader's sensitivity to the factors that affect health (such as stress). A sample item is “My supervisor immediately notices when something is wrong with my health.” Health behaviors include providing healthy working conditions, obtaining employees' health information, and encouraging employees to practice healthy behaviors. A sample item is “My supervisor invites me to inform him/her about health risks at my workplace.” The measurement scale developed by Franke et al. (2014) has been widely used in empirical research and has been found to have good reliability and validity (Kranabetter and Niessen, 2017; Horstmann, 2018b; Kaluza et al., 2018; Köppe et al., 2018).

Third, researchers can revise other construct scales to measure health-promoting leadership. For example, Jiménez et al. (2016) broadened the dimensions of the Areas of Work Life Scale (Leiter and Maslach, 1999) by adding health awareness as a core dimension. The revised scale has 7 dimensions and 21 items. Among them, the α coefficient of the health awareness dimension is 91, and a sample item is “Employee health is highly valued.” The remaining dimensions are workload (α =0.76; sample item: “Work does not significantly affect private life”), work control (α = 0.75; sample item: “At work, autonomous and independent action can be taken”), reward (α = 0.83; sample item: “All contributions are acknowledged”), work community (α = 0.86; sample item: “Work colleagues support each other”), fairness (α = 0.79; sample item: “All employees are treated in a fair manner”), and employee–organization value consistency (α = 0.75; sample item: “The employees share the company's values”). Subsequently, this scale was used in a series of empirical studies (Jiménez et al., 2017a; Bregenzer et al., 2019).

Exploring the structure of health-promoting leadership can be challenging due to the ongoing controversy about the most appropriate measurement approach for this structure. As a result, the content and focus of the currently available measurement tools differ dramatically, which in turn limits the development and application of health-promoting leadership measurements. Moreover, most of these measurements have been revised from or adopted based on existing measurement tools (e.g., Areas of Work Life Scale) from related research fields and, therefore, ignore the unique aspects of health-promoting leadership. Thus, the development of measurement scales that have high reliability and validity and that would be recognized by most scholars remains an unmet need.

## Research Framework for Health-Promoting Leadership

**Figure 2:**
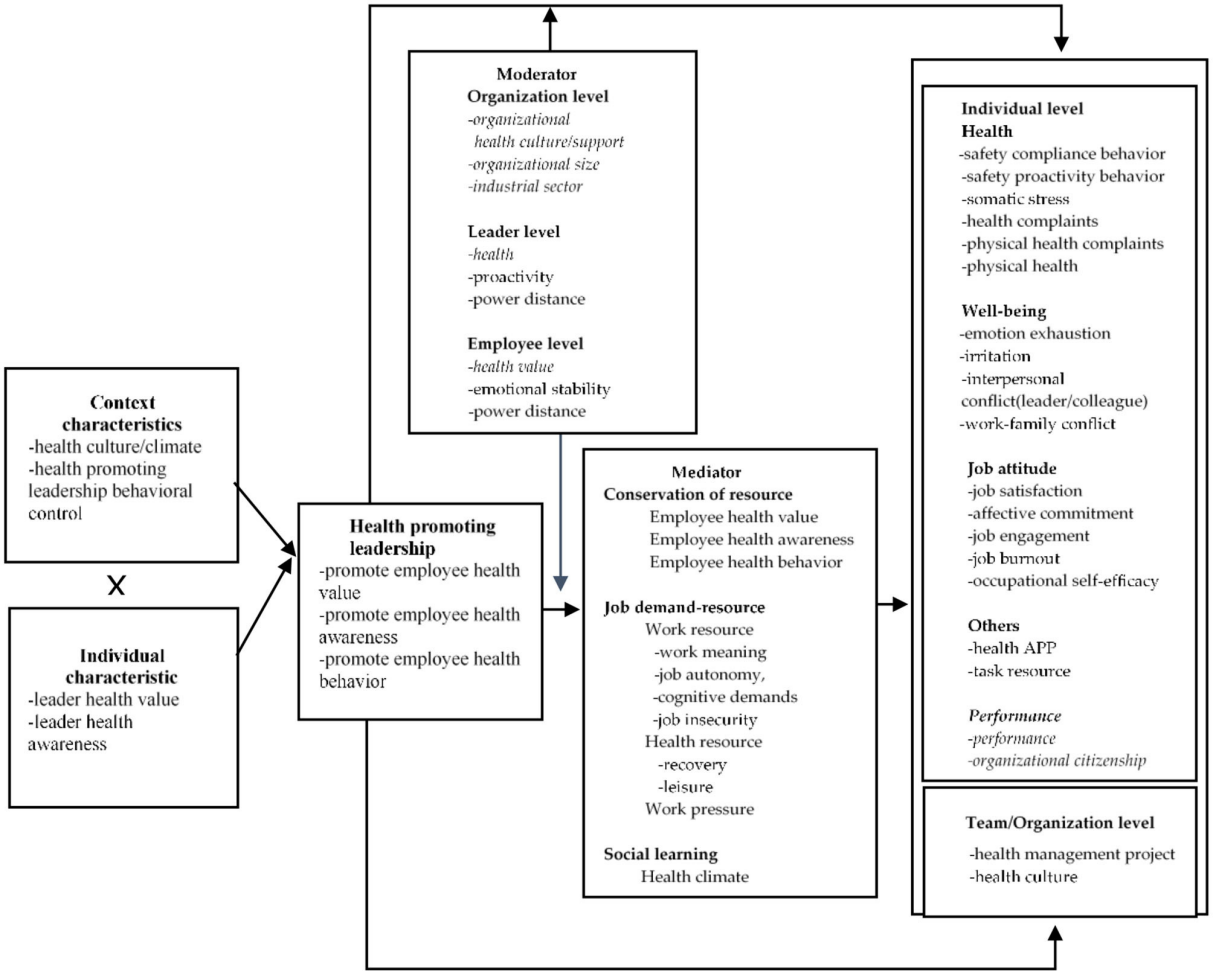
Research framework for health-promoting leadership. Italics indicate future research.

The research framework for health-promoting leadership (shown in Figures 1, 2) that we developed includes the consequences, mechanisms, boundary conditions, and antecedents of this type of leadership. Health-promoting leadership plays an important role in employee health and well-being, as well as in organizational sustainable development. The consequences of such leadership can be separated into those occurring at the individual level vs. at the team or organizational level. Most studies have focused on employee health and well-being at an individual level. Given that the health-promoting leadership is positively related to employee job satisfaction, job engagement, and affective commitment (Gurt et al., 2011; Horstmann, 2018b), and negatively related to job burnout and interpersonal conflict (Jiménez et al., 2017a; Bregenzer et al., 2019), we can speculate that health-promoting leadership may improve performance at the employee, team, and organizational levels.

Wegge et al. (2014) identified five pathways by which leadership behavior can affect employee health: (1) directly promote (or damage) employee health; (2) promote (or damage) employee health by designing work systems that reduce the pressure on employees and increase their resources; (3) play a moderating role in the relationship between environmental factors and employee health; (4) cultivate a healthy climate and build a common identity; and (5) act as a role model.

Based on Wegge et al. (2014) research and other studies on health-promoting leadership, we propose our own model of how health-promoting leadership affects employee health. We suggest that health-promoting leadership influences employees in both direct and indirect ways. Consistent with Wegge et al. (2014), direct influence implies that health-promoting leadership is employee oriented and directly affects employees' health and well-being. In addition, leaders' behavior may affect employee health in four indirect ways, each underpinned by a different theory from the social sciences. First, based on conservation of resources theory, health-promoting leadership affects employee health and well-being by providing internal resources and external resources that may change employees' health values, health awareness, and health behaviors (Franke et al., 2014). Second, the job demands–resources model suggests that health-promoting leadership would increase employees' work resources, reduce employees' work demands by changing employees' perceptions of their job characteristics, and ultimately affect employee health and well-being (Winkler et al., 2014; Bregenzer et al., 2019). Third, consistent with social learning theory, health-promoting leaders create a culture and climate that promote health by role modeling the desired behaviors, which in turn affects employee health and well-being (Milner et al., 2015; Liu, 2016). Fourth, social exchange theory implies that health-promoting leadership may affect employee health by making employees explicitly perceive the organization's commitment to their health, which in turn encourages employees to demonstrate the expected behaviors (e.g., participate in healthy practices) (Barrett et al., 2005).

The existing research on health-promoting leadership mentions the intermediate pathway posited by social exchange theory, social learning, conservation of resources theory, and the job demands–resources model. Following the Wegge et al. (2014) study, future research may explore the mediation pathway through social identity perspective and moderator pathway that health-promoting leadership could work to enrich studies.

Contingency factors that may moderate these effects can be divided into three levels: employee, leadership, and organization. Current research has tended to focus on the employee and leadership levels. At the employee level, when employee power distance orientation is high, health-promoting leadership can significantly increase the social resources that employees perceive they could receive (Bregenzer et al., 2019). Power distance orientation refers to the extent to which an individual expects and accepts an unequal power distribution (Dorfman and Howell, 1988); that is, it indicates the degree to which an individual accepts what and how he or she is told something by a higher-status person (Madlock, 2012). Compared with high power distance orientation employees, low power distance orientation employees tend to be more strongly affected by their leaders.

Employees' emotional stability may also strengthen the impacts of health-promoting leadership. For example, Bregenzer et al. (2019) found that when employees' emotional stability is low, health-promoting leadership may boost employees' social resources.

At the leadership level, a high level of leadership initiative (Horstmann, 2018a) enhances the effects of health-promoting leadership. In contrast, when leaders' power distance orientation is high, the impacts of health-promoting leadership decrease (Winkler et al., 2014).

To date, no studies have explored moderators on the organizational level. Future research, therefore, might investigate organization size or industrial sector as potential factors affecting health promotion within the workplace. Organization size is an important context variable that can affect the function of leadership. Research has found that the impact of leaders may decrease in larger organizations, whereas that impact may increase in smaller organizations (Nahavandi and Malekzadeh, 1993; Koene et al., 2002). In terms of type of business, the public sector has some distinct differences from the industrial sector (Vanhala and Stavrou, 2013). For example, organizations in the public sector often pursue political objectives, whereas private-sector businesses pursue the singular goal of making a profit (Boyne, 2002). Moreover, leaders in the public sector have less ability to reward employees compared with their counterparts in the private sector (Anderson et al., 2005). Based on these characteristics, we speculate that there may be differences in health-promoting leadership and employee well-being in the public sector vs. the industrial sector. Future research could explore these relationships in more depth.

The antecedents of health-promoting leadership can provide effective guidance for the development of such leadership. At present, few research studies have addressed this aspect. The existing studies have found that a positive organizational health climate enhances leaders' health awareness, prompting them to display more health-promoting behaviors (Kaluza et al., 2018). In contrast, greater organizational control over health-promoting behaviors tends to inhibit leaders from emphasizing behaviors that promote employee health (Turgut et al., 2019). In terms of individual characteristics, leaders' health awareness and health values have a positive effect on health-promoting leadership.

### The Consequences of Health-Promoting Leadership

Scholars focused on investigating the impacts of health-promoting leadership have examined both individual health and organizational health capabilities. Specifically, at the individual level, the existing research has mostly adopted cross-sectional study designs and examined the effects of health-promoting leadership on outcomes such as employees' health, well-being, and job attitude.

#### Health

Evidence suggests that health-promoting leadership plays an important role in several employee health-related outcomes: safety compliance behavior, safety proactivity behavior, somatic stress, health complaints, physical health complaints, and physical health. For example, Nielsen et al. (2019) indicated that health-promoting leadership could be regarded as a resource in directing employees' health safety behaviors, and may enable (geographically) distributed workers to engage in safety compliance and safety proactivity behaviors. Franke et al. (2014) argued that health-promoting leadership can be seen as a staff-care leadership (i.e., demonstrating concerns about employee health), and as positively related to employees' physical health and negatively related to employees' health complaints, including their physical health complaints. Rigotti et al. (2014) found that health-promoting leadership can reduce somatic stress. When Bregenzer et al. (2019) examined the relationship between health-promoting leadership and employees' health-related resources, they found that such leadership has a positive relationship with employees' task resources and social resources, which ultimately influences employees' health. In addition, Gurt et al. (2011) found that health-promoting leadership is positively related to the psychological climate for health perceived by employees.

When they are under constant strain, employees often experience symptoms such as headache, loss of appetite, and sleep disturbances (Franke et al., 2014). These kinds of health complaints are an important predictor of chronic diseases such as gastrointestinal diseases, cardiovascular diseases, and diabetes (Köppe et al., 2018). Studies examining the relationship between health-promoting leadership and such outcomes have relied on data taken from multiple industries and organizations, such as manufacturing, communications, healthcare, education, consulting, and government departments in Austria, China, German. and Slovenia, and have mainly targeted low-skilled employees (Winkler et al., 2014), geographically distributed employees (Nielsen et al., 2019), and government officials (Franke et al., 2014). Ultimately, they have shown that health-promoting leadership is positively related to employee health.

#### Well-Being

Well-being may be divided into psychological, social, and physical well-being.^1^ Exhaustion could be regarded as a central variable to measure employee psychology well-being (Liu and Jia, 2020). As Winkler et al. (2014) found that health-promoting leadership decreases employees' emotional exhaustion. Employees have three basic psychological needs—the need to belong, the need to be seen as competent, and the need for autonomy—that promote their psychological well-being (Liu and Jia, 2020). Health-promoting leadership cares about employees' health and well-being; improves their health awareness, health knowledge, and skills; and provides employees with adequate work resources and sufficient job autonomy and job control (Bregenzer et al., 2019). Collectively, these daily leadership behaviors may satisfy employees' psychological needs and decrease their exhaustion.

Moreover, research has found that health-promoting leadership is negatively related to irritation (Franke et al., 2014). Irritation is generally viewed as an outcome of poor psychological well-being, signaling the negative results of work cognition and emotional strain (Franke et al., 2014). It manifests as a habitual irritability reaction, so that it is difficult for the employee to relax after work (Gurt et al., 2011).

Both Horstmann (2018b) and Milner et al. (2015) showed that health-promoting leadership promotes good relationships between employees and their leaders and colleagues. Such leadership takes responsibility for employees' health and demonstrates care for their health needs, thereby signaling to employees that the organization has concern for them and values them. In return, employees may feel obligated to pay back these “favors” to the organization. For example, employees may be more inclined to treat their colleagues well and maintain high-quality relationships with them. Health-promoting leadership may also directly provide employees with social resources, which benefits employees as social capital and enhances their social resources. Finally, Franke et al. (2014) showed that health-promoting leadership is negatively related to employee work–family conflict.

Studies of the relationship between health-promoting leadership and employee well-being have involved various types of organizations and often used large sample sizes. For example, Gurt et al. (2011) studied 1,969 employees from a German Taxation Office. Milner et al. (2015) surveyed 11,472 employees from 71 companies in South Africa. Horstmann (2018b) studied 861 employees in 28 elderly institutions in Germany. Collectively, their research results clearly establish that health-promoting leadership is positively related to employee well-being.

#### Job Attitude

Research has shown that health-promoting leadership works through both direct paths (i.e., work engagement) and indirect paths (i.e., job satisfaction, organizational affective commitment, job burnout, and occupational self-efficacy). For example, Milner et al. (2015) found that health-promoting leadership supports workplace health programs, with employees then perceived the company as being committed to their health, which ultimately increases employees' job satisfaction. Horstmann (2018b) found that health-promoting leadership is linked to increased social resources and subsequently enhances organizational affective commitment. Kaluza et al. (2018) found that such leadership is positively related to work engagement.

Horstmann (2018b) noted that health-promoting leadership is negatively related to employee job burnout through employee self-care (e.g., taking care of the employee's own health). Jiménez et al. (2017a) also indicated that this kind of leadership may decrease employees' risk of job burnout. Specifically, health-promoting leadership enhances employees' resources by changing workpace conditions, so that employees are less prone to burnout. In addition, Rigotti et al. (2014) demonstrated that health-promoting leadership has a positive relationship with employees' occupational self-efficacy through job autonomy, work meaning, cognitive demands, and job insecurity across 8 and 22 months.

#### Health Management Culture and Practices at the Organizational Level

Organizational health culture and organizational health practice projects are important components of organizational health management (Eriksson et al., 2012; Skarholt et al., 2016). We found three studies that focus on health management culture and practices. Gurt and Elke (2009) found that health-promoting leadership is positively related to organizational health culture, while general leadership behavior is unrelated to it; thus, supporting the organizational health culture is a unique function for health-promoting leadership. Adopting a case study design, Eriksson et al. (2010) found that health-promoting leadership is positively related to the effectiveness of health-promoting projects. Whiteman et al. (2011) studied a health management project within the U.S. Navy focused on quitting smoking and alcohol use, showing that the positive outcome was directly related to the successful implementation of the project in which leadership was engaged.

#### Other Outcome Variables

Health-promoting leaders often use health apps and are positively related to health-related resources. For example, Bregenzer et al. (2017) showed that such leaders use health-promoting apps more often than non-health-promoting leaders, which suggests that health-promoting leaders are more aware of their health condition and more likely to communicate with others on health-related issues. Bregenzer et al. (2019) also found that health-promoting leadership is positively related to health-related resources, such as task resources.

### Mediational Mechanisms of Health-Promoting Leadership

Health-promoting leadership may affect employees' health and well-being both directly and indirectly. Notably, when it takes the form of staff-care leadership (e.g., demonstrating care for employees' health) or directly provides health resources to employees, health-promoting leadership may have a direct influence on employees. In other instances, it may impact employees indirectly. In this section, we summarize the core mechanisms underlying the distinct effects of health-promoting leadership: conservation of resources theory, job demands–resources theory, social exchange theory, and social learning theory (see Table 3). Based on the “content analysis method” suggested by the classic literature (Gardner et al., 2011), we systematically organized our research on the theme of health-promoting leadership spread over the period 2005–2020. We studied a total of 50 papers, including 5 studies that adopted conservation of resource theory, 7 that focused on social learning theory, 5 that used job demands–resources theory, and 2 that used social exchange theory. In terms of overall trends, conservation of resource theory and social learning theory were mostly adopted in studies over the 2018–2019 period. The job demands–resources model was mostly adopted over the period 2017–2018.

**Table 3:** Summary of theory.

**Theory**	**Mechanisms**	**Outcomes**	**References**
Conservation of resource theory	Promote employee health by affecting employees' health awareness, values, and behavior (resources-related health)	Safety, physical health, mental health, work well-being, psychological well-being, and life well-being	Franke et al., 2014; Liu, 2016
Job demands–resources model	Change employees' perception of job characteristics (reduce job demands, increase job resources), thereby reducing employee work pressure and ultimately improving employee health	Safety, physical health, mental health, work well-being, psychological well-being, and life well-being	Winkler et al., 2014; Jiménez et al., 2017a,b; Kaluza et al., 2018; Vincent-Höper and Stein, 2019
Social exchange theory	Make employees feel their leader is concerned about their health, so employees will develop a sense of obligation to pay back the organization, and therefore exhibit a series of attitudes or behaviors expected by the organization	Work well-being, psychological well-being, and life well-being	Milner et al., 2015
Social learning theory	As role models for employee behavior, leaders encourage employees to actively imitate them; this learning process seeks to cultivate a healthy climate and promote employee health	Safety, physical health, mental health	Gurt and Elke, 2009; Gurt et al., 2011; Skarholt et al., 2016

#### Conservation of Resources Theory

Conservation of resources (COR) theory states that individuals continually strive to acquire, store, and protect resources that are valuable to themselves (Hobfoll, 1989; Gardner et al., 2011). Any potential or actual loss of resources, therefore, will create individual pressure. Moreover, individuals with resources find it easier to obtain additional resources and are less susceptible to resource losses; conversely, individuals lacking resources are more susceptible to resource losses and find it more difficult to obtain resources (Hobfoll et al., 2018).

Health-promoting leaders provide employees with plenty of resources that could promote their health (e.g., health knowledge, health-promoting activities). Those employees who receive health resources are expected to change their feelings, their thinking, and their behavior patterns in a positive direction in terms of health, which ultimately affects their physical and mental health (Eriksson et al., 2011; Franke et al., 2014). Research based on COR theory has generally adopted employees' health awareness, health values, and health behaviors as mediating variables. For example, Franke et al. (2014) found that health-promoting leaders can change employees' health awareness, health values, and health behaviors by providing health resources to employees, and ultimately reduce employees' health complaints and irritations.

#### Job Demands–Resources Model

The job demands–resources (JD-R) model states that excessive work requirements consume staff resources, and a lack of work resources increases the difficulty of completing their work for employees (Demerouti et al., 2001). Both processes increase work pressure, which ultimately jeopardizes the effectiveness of employees and organizations (Bakker and Demerouti, 2017). Similar to COR theory, the JD-R model emphasizes the important effects of work resources for employees' physical and mental health; that is, having sufficient job resources can alleviate the pressure of work requirements on employees (Winkler et al., 2014; Bregenzer et al., 2019).

Research based on the JD-R model generally takes job demands and job resources as mediating variables. For example, Jiménez et al. (2017a) found that health-promoting leadership reduces employees' health stress and job burnout by increasing health resources (i.e., recovery, leisure time, psychological resources, and work resources). Vincent-Höper and Stein (2019) revealed that health-promoting leadership increases employees' job resources and reduces their job demands by shaping employees' psychological job characteristics (i.e., work clarity, work control, and work feedback); in doing so, it ultimately increases work engagement, well-being, and occupational self-efficacy, and reduces irritation and emotional exhaustion. Similarly, Winkler et al. (2014) found that health-promoting leaders provide valuable resources such as social support, task-related communication, individual consideration, and positive feedback to employees, thereby increasing their job satisfaction and reducing their emotional exhaustion and health complaints.

#### Social Exchange Theory

Social exchange theory explains how people form mutually beneficial relationships through exchange activities and produce expected results for both sides (Cropanzano and Mitchell, 2005). When leaders provide valuable resources, employees will feel an obligation to perform in such a way as to give back to the organization. In our context, organizations and employees will forge a mutually beneficial relationship on health issues: On the one hand, healthy employees will increase organizational productivity and reduce medical expenses; on the other hand, health is a fundamental need of employees. Health-promoting leadership builds a healthy workplace for employees and satisfies their health needs, which sends a signal that the organizations truly care about their employees. Employees, in turn, feel a sense of obligation to repay the organizations, which positively affects their individual attitudes, such as job satisfaction and organizational commitment. For example, Milner et al. (2015) found that health-promoting leaders engage in management projects that promote health by implementing company policies aiming to promote employee health. Employees, in turn, perceive that the organization is committed to the betterment of their health. This generates employees' willingness to pay back the organization, thereby increasing their job satisfaction, reducing their job burnout, and decreasing their interpersonal conflicts.

#### Social Learning Theory

Social learning theory explicates how the interaction of cognition, behavior, and environment influence individual behavior during the learning process (Bandura et al., 1977). Leadership that represents a healthy physical and mental state is attractive to employees; employees are prone to imitating these kinds of leader health behaviors, thereby generating a culture and climate that promotes health, and resulting in increased employee health (Gurt et al., 2011). Kranabetter and Niessen (2017) suggest that health-promoting leaders can act as role models in four ways: (1) encourage employees to demonstrate health-promoting behaviors at work; (2) encourage employees to imitate key behaviors that promote health; (3) encourage employees to gain and reflect on health experiences; and (4) encourage employees to create a new healthy workplace. Such leader role modeling is expected to cultivate a culture and climate that promotes employee health. For example, Gurt and Elke (2009) found that health-promoting leadership reduces work pressure on employees by creating an organizational health culture.

### The Antecedents of Health-Promoting Leadership

The antecedents of health-promoting leadership include individual characteristics of the leader (e.g., health values, health awareness) and context characteristics (e.g., health culture, organizational climate, and health-promoting leadership behavioral control). Franke et al. (2014) proposed that leaders' health awareness, health values, and health behaviors are important antecedents for health-promoting leadership. Unfortunately, these relationships have not been tested empirically.

The presence of an organizational health culture enables leaders to exhibit behaviors that promote employee health. Organizational health-promoting behavioral control refers to leaders' perception that their organization restricts their behavior in promoting employee health (Turgut et al., 2019). Using a sample of German managers in an automobile manufacturing plant, Turgut et al. (2019) found that organizational health culture has a positive effect on health-promoting leadership, whereas health-promoting leadership behavioral control may inhibit leaders from explicitly modeling behaviors that promote employee health.

## Future Research

Health-promoting leadership has important practical implications for the sustainable development of organizations. Research in this area remains in its infancy, and the unmet needs have a wide scope. For example, a widely accepted measurement scale is needed. Research methods, research levels, and outcomes need to be constantly enriched. Due to the increasing emphasis on employees' health and well-being in leadership research and practice, additional research in this area is clearly required. In this section, we identify six issues for further research on health-promoting leadership.

First, in regard to the research level, future studies could explore the effects and mechanisms of health-promoting leadership at both the team level and the organizational level. Current research has generally focused on the individual level (Eriksson et al., 2011; Liu, 2016; Jiménez et al., 2017b), with only a few empirical studies examining the effects of health-promoting leadership at the team or organizational level (Akerjordet et al., 2018). Given that the goal of health-promoting leadership is to achieve sustainable development of the organization (Jiménez et al., 2017b), it is important to explore health behaviors and performance at the team and organization levels. In addition, the mechanisms of leadership would be different at the individual and team levels (Wang and Howell, 2012). For example, Wu et al. (2010) found that individual-focused leadership reduces team effectiveness due to team member differences both in leadership identification and in self-efficacy. Conversely, group-focused leadership has a positive impact on team effectiveness because it promotes both team identity and collective effectiveness (Wu et al., 2010). Related possible research questions include: Is there a difference in the mechanism of health-promoting leadership at the individual, team or organizational level? What role does health-promoting leadership play at the team and organizational levels? Which mechanisms of health-promoting leadership operate at the team or organizational levels? Such questions could be explored through follow-up research to establish a greater explanatory system of theoretical models.

Second, future studies can elaborate the effects of health-promoting leadership on employee performance. The current research has focused on employees' health and well-being, but largely ignored their work performance. Previous studies have found that employees' physical health, mental health, and well-being have positive effects on organizational performance (Wu et al., 2010; Nielsen et al., 2019). In addition, future research could explore the relationship between health-promoting leadership and performance-related work behaviors, such as organizational citizenship behaviors or counterproductive behaviors. Social exchange theory suggests that health-promoting leaders might build high-quality exchange relationships with employees by caring about their health (Reader et al., 2017), and employees may then engage in organizational citizenship behaviors to repay the organization. Meanwhile, COR theory implies that health-promoting leaders could provide health resources to employees and reduce their resource consumption at work (Bakker and Demerouti, 2007), thereby strengthening employee self-control and in turn reducing counterproductive behavior (Marcus and Schuler, 2004). Future research can further explore these relationships to deepen the understanding of the role that health-promoting leadership plays.

Third, future work can examine the impacts of health-promoting leadership on leaders themselves. This type of leadership may have a “double-edged sword” effect on leaders, and future research could explore it further. On the one hand, in the process of affecting employees' health and well-being, leaders may increase their health awareness, acquire more knowledge about health, and increase their own positive health behaviors, which could boost resources for these leaders. On the other hand, faced with the responsibility of managing employee health, health-promoting leaders may spend more time and energy on ways to accomplish this goal, thereby consuming more of their personal resources. Future research could examine the consequences and boundary conditions of health-promoting leadership in terms of resource gain or resource loss.

Fourth, in terms of the mechanism, future research could explore additional moderators at the organization, leader, and employee levels. The existing research has examined the boundary conditions of health-promoting leadership from the perspectives of leaders and employees, such as leadership power distance orientation (Winkler et al., 2014) and initiative (Horstmann, 2018a), and employee power distance orientation and emotional stability (Montano et al., 2017). In regard to new moderators, at the organizational level, organizational health culture and organizational health support might be examined, among other possibilities. Organizational health culture establishes norms for leaders and employees (Barrett et al., 2005), which influences the effectiveness of interactions between leaders and employees. Therefore, organizational health culture may enhance the effects of health-promoting leadership. Similarly, organizational health support would provide employees with more functional support and emotional support (Eriksson et al., 2011), reduce resource depletion among health-promoting leaders, and make health-promoting leadership more effective. From the leader perspective, leader health status is another important factor that influences the sustainability of health-promoting leadership. The healthier the leader, the more effective the health-promoting leadership will be (Nielsen and Taris, 2019). At the individual level, employees with greater health values are more affected by health-promoting leadership behaviors (Franke et al., 2014), which may enhance the effectiveness of health-promoting leadership on employees.

Fifth, in terms of research methods, the current research has mainly relied on survey methods, and most published work consists of cross-sectional studies. Leaders may exhibit different behaviors at different times, and a cross-sectional study design has difficulties in capturing the full complexity of leadership behaviors. It may be necessary to adopt the experience sampling methodology (ESM; Bolger and Laurenceau, 2013) to examine the dynamic process by which leadership behavior affects employee health. In addition, a cross-sectional design makes it more difficult to clarify the causal relationship between health-promoting leadership and employee health. There may also be reverse and reciprocal relationships between them. In the future, longitudinal studies can be used to identify causal effects (Winkler et al., 2014; Reader et al., 2017). This study design can also reveal the effects of short-term and long-term employee health and happiness, and provide richer and more nuanced information on employee health. It is worth noting that the most important role of health-promoting leadership for employees relates to employee health, but the existing research lacks an objective way to measure health in this context. Future research might use objective health indicators, such as blood pressure and skin electricity for this purpose (Winkler et al., 2014; Reader et al., 2017). Warnsley (2015) also recommends using incidence/prevalence of certain diseases to test the impact of health-promoting leadership on employee health—for example, anxiety, depression, high blood pressure, diabetes, and cancer.

Finally, future research could investigate the training effects of health-promoting leadership. Rigotti et al. (2014) found that a training program could develop health-promoting leadership and have substantial effects on employees' health and well-being. Future studies could utilize an experimental design, including intervention and control groups, to see if interventions (e.g., training, coaching) change health-promoting leadership, with this change subsequently being seen at the employee (or group) level.

## Conclusion

As the health of employees has become an increasingly prominent concern, researchers have gradually honed in on the need to conduct health research in the workplace. From the perspective of theory development, the concept of health-promoting leadership may enrich the leadership research. From the perspective of management practice, the existing research provides valuable guidance for organizations' sustainable development and delineates effective paths for leaders to follow. In this paper, we have systematically sorted out the concept, structure and measurement of health-promoting leadership. We have also explained the effects of health-promoting leadership based on COR theory, the JD-R model, social exchange theory, and social learning theory. Finally, we have analyzed the antecedents of health-promoting leadership in both the individual and organizational contexts.

By systematically reviewing the advancements in health-promoting leadership research, our work makes three contributions to the field. First, our systematic review of the research literature on health-promoting leadership elucidates the concept of health-promoting leadership, structure and measurement, consequences, theoretical explanation, antecedents, and future research prospects, providing research directions and promising paths for further research on health-promoting leadership.

Second, we define the concept of health-promoting leadership from a general perspective and a specific perspective, thereby clarifying the essence of health-promoting leadership—a point that has remained largely a puzzle in previous studies.

Third, by reviewing previous studies and combining the extant work with forward-looking trends, we are able to propose overlooked research topics. For example, we recommend that researchers consider the level of research, examine the effects of health-promoting leadership on performance and leaders themselves, and seek out richer research methods (e.g., using experimental research designs to reveal the effects of health-promoting leadership).

## Author Contributions

LY is responsible for the draft paper. PL is responsible for the review and editing. HW is responsible for final paper review and editing. All authors have read and agreed to the published version of the manuscript.

## Conflict of Interest

The authors declare that the research was conducted in the absence of any commercial or financial relationships that could be construed as a potential conflict of interest.

## Footnotes

^1^Physical well-being is discussed in section “Health.”
